# Working Memory Load Affects Processing Time in Spoken Word Recognition: Evidence from Eye-Movements

**DOI:** 10.3389/fnins.2016.00221

**Published:** 2016-05-19

**Authors:** Britt Hadar, Joshua E. Skrzypek, Arthur Wingfield, Boaz M. Ben-David

**Affiliations:** ^1^Baruch Ivcher School of Psychology, Interdisciplinary Center HerzliyaHerzliya, Israel; ^2^School of Psychological Sciences, Tel-Aviv UniversityTel Aviv, Israel; ^3^Volen National Center for Complex Systems, Brandeis UniversityWaltham, MA, USA; ^4^Rehabilitation Sciences Institute, University of TorontoToronto, ON, Canada; ^5^Department of Speech-Language Pathology, University of TorontoToronto, ON, Canada; ^6^Toronto Rehabilitation Institute, University Health NetworksToronto, ON, Canada

**Keywords:** working memory, speech perception, word recognition, eye-tracking, visual world paradigm

## Abstract

In daily life, speech perception is usually accompanied by other tasks that tap into working memory capacity. However, the role of working memory on speech processing is not clear. The goal of this study was to examine how working memory load affects the timeline for spoken word recognition in ideal listening conditions. We used the “visual world” eye-tracking paradigm. The task consisted of spoken instructions referring to one of four objects depicted on a computer monitor (e.g., “point at the candle”). Half of the trials presented a phonological competitor to the target word that either overlapped in the initial syllable (onset) or at the last syllable (offset). Eye movements captured listeners' ability to differentiate the target noun from its depicted phonological competitor (e.g., candy or sandal). We manipulated working memory load by using a digit pre-load task, where participants had to retain either one (low-load) or four (high-load) spoken digits for the duration of a spoken word recognition trial. The data show that the high-load condition delayed real-time target discrimination. Specifically, a four-digit load was sufficient to delay the point of discrimination between the spoken target word and its phonological competitor. Our results emphasize the important role working memory plays in speech perception, even when performed by young adults in ideal listening conditions.

## Introduction

Although, seemingly performed without effort, understanding speech is a complex task (Pollack and Pickett, 1963; Lindblom et al., 1992; Wingfield et al., 1994; Murphy et al., 2000). During the process of spoken-word recognition, listeners must simultaneously retain and process the context of the sentence, keep the previous spoken words activated, segregate the speech signal from noise, and inhibit the potential activation of alternatives for the spoken word (e.g., phonetic or semantic). All of these operations might draw on the same resources necessary for speech processing and, as a result, may compromise recognition. The current study presents, to the best of our knowledge, the first examination of the impact of working memory load on the *online* processing of a single spoken word in ideal listening conditions. For this purpose, we examined eye-movements using the visual world paradigm (Tanenhaus et al., 1995) to reveal listeners' timeline for recognition of target words.

## Spoken-word recognition

Most current models of speech perception are activation-competition models, in which auditory input activates a set of lexical candidates, which then compete for the highest level of activation. Lexical access is a product of the integration of bottom-up and top-down processes (e.g., see the Cohort model, Marslen-Wilson, 1987, 1990; TRACE model McClelland and Elman, 1986). Bottom-up information is supplied by the acoustic-phonetic features of the sound wave, while the top-down information consists of the semantic and syntactic information related to the input (Rönnberg et al., 2013). As the acoustic signal unfolds in time, an analysis of the signal features allows the system to match phonetic cues to word forms in the mental lexicon. For example, hearing the initial phoneme /kæ/ will activate the words *candy, candle, cannon, camel*, etc. As the utterance of the word progresses to include more phonemes, irrelevant alternatives are inhibited until the listener reaches the isolation point—the point in time at which the target word is distinguished from its alternatives. The continuous uptake of speech sounds from the unfolding spoken word also activates offset-sound sharing alternatives that act as phonological competitors e.g., *candle*—*sandal* (Wayland et al., 1989; Wingfield et al., 1997; Luce and Pisoni, 1998; Sommers and Amano, 1998). These alternatives activated at the end of the word, were also found to delay the isolation point (Allopenna et al., 1998), as lexical access takes place continuously. This offset-overlap effect was noted more strongly in populations with reduced working memory capacity (e.g., older adults, Ben-David et al., 2011). For example, if the onset of the word was not enough to lead to an isolation point, the additional information at the end of the word can add alternatives and thus further delay this point.

Studies of speech perception have primarily focused on accuracy-based assessments to provide information about the overall integrity of speech perception. Such off-line measures, however, make it difficult to determine the specific processes underlying this accuracy. To overcome this limitation, we investigated linguistic processing using the “visual world” eye-tracking paradigm (Tanenhaus et al., 1995). In this paradigm, listeners are asked to follow spoken instructions referring to objects depicted on a computer monitor (the “visual world”). For example, participants might hear the phrase, “*point at the candle*,” and simultaneously see a display containing four pictures, each representing a word: *candle* (target)*, sandal* (offset-competitor)*, finger*, and *zebra* (unrelated nouns). As the listeners hear the instructions and the unfolding sound of the object's name, their eye-gaze data are time-locked with what is being heard on a moment-to-moment basis. With this, we were able to record where a person is looking on a visual display, how long their eye dwelled on a location, and the rate and order in which their gaze moved to other locations. To illustrate the method, consider our example of a listener listening to the phrase, “*point at the candle*,” where both a candle and a sandal are depicted on the display. We track, in real-time, as the listener shifts his or her focus between *candle* and *sandal*, which share the terminal phoneme /də1/. One can record, with millisecond accuracy, whether focus on the target, *candle*, is delayed due to competing activation of the offset competitor, *sandal*, as reflected by the listener's gaze pattern. In this way, eye movements can reveal the point at which listeners are able to isolate a target word from its competitor.

The visual world paradigm can also gauge what factors might either impede or facilitate spoken word processing, and to what extent. For example, the paradigm has been used successfully to test the impact of stream segregation of a spoken word from a noisy background (Ben-David et al., 2011) as well as from competing speech (Helfer and Staub, 2014). In the current study, we used this paradigm to investigate the role of working memory load. Listeners were asked to recognize the spoken word and touch the relevant pictogram, while retaining in memory spoken digit(s) presented at the beginning of a trial.

## Speech processing and working memory

Working memory is a fundamental cognitive mechanism that allows active maintenance and manipulation of a limited amount of information (Luck and Vogel, 1997; Awh et al., 2007). Many complex cognitive tasks, including understanding speech, rely on working memory support (Baddeley, 1992; Luck and Vogel, 2013). Because working memory capacity is limited, any increase in demands on working memory should decrease the capacity available to actively maintain and process additional information.

In experimental settings, a dual task paradigm can reveal the toll individuals pay when resources are occupied by a concurrent task (Pashler, 1994). Participants in the dual task paradigm are asked to perform two simultaneous tasks. As the demands of the primary task increase, the available resources for the secondary task decrease (Sarampalis et al., 2009; Tun et al., 2009; Campana et al., 2011). Thus, the extent of the decrease in performance in the secondary task can point to the degree of resources demanded by the primary task (Kerr, 1973).

It has been argued that differences in working memory capacity may stem from differences in the efficiency of inhibiting irrelevant information. Vogel et al. (2005) found that individuals with low working memory capacity find it harder to inhibit irrelevant information than do high-capacity individuals (see also Lash et al., 2013). Similarly, working memory capacity predicts participants' ability to inhibit irrelevant distractors in a Flanker task (Heitz and Engle, 2007). Awh and Vogel (2008) view working memory as responsible for inhibiting irrelevant sensory information, naming it as the “bouncer in the brain.” Lavie et al. (2004) found that an increase in working memory load increases distractor interference in the visual domain. They suggested a working memory based cognitive control mechanism that decreases interference from distractions. Once this control mechanism is occupied by a task that demands working memory resources, inhibition efficiency is decreased in any other task. In speech recognition, an increase in working memory demands might be reflected by a decrease in the ability to inhibit the activation of word alternatives.

Another approach that considers working memory as an important player in the speech perception process is the *Ease of Language Understanding model* (ELU; Rönnberg, 2003; Rönnberg et al., 2008, 2013). According to the ELU model, the language input receives implicit processing at the episodic buffer, and is then compared to phonological information stored in long-term memory. This model suggests that this “implicit” process is completed rapidly, with little or no draw on resources. However, if a mismatch occurs between the signal and its corresponding representation in long-term memory, slower, resource-demanding “explicit” processing is required. Thus, when the competition increases between the bottom-up sound information and possible word alternatives, resources are recruited for “explicit” speech processing and successful word identification will take longer to complete.

Although, in the discussions that follow we contrast implicit vs. explicit processing following Rönnberg et al. (2013), we recognize that these terms may be more accurately seen as denoting two ends of a continuum, reflecting degrees of resource demands for success (see the discussion in Wingfield et al., 2015).

## Current study

The goal of the current study was to examine the extent to which working memory load affects the timeline for the processing of a single spoken word. As a first step, we adapted the visual world paradigm (Tanenhaus et al., 1995) to Hebrew and validated it. Two types of sound-sharing competitors were presented on different trials, onset- and offset-overlap. The target words and their phonological competitors were matched on linguistic characteristics, such as frequency, familiarity, and number of syllables. The corresponding pictograms were matched for recognizability and visual saliency. Next, we tested online recognition of a spoken word using the visual world paradigm, with two levels of working memory pre-load: high vs. low load. In the beginning of each trial, either one spoken digit (low load) or four spoken digits (high load) were presented. Participants were asked to retain the digit(s) while performing the spoken word recognition task. Once they had indicated their recognition of the spoken word (by touching the correct pictogram), they were asked to verbally recall the digit(s). By using eye-tracking with high-resolution data in the millisecond level, our goal was to reveal the exact timeline of word processing and the factors that may facilitate or impede each stage of the process until recognition occurs.

Applying the ELU model to the visual world paradigm described above yields several predictions. Mainly, as the competition increases between top-down and bottom-up information, there will be a shift from an implicit to an explicit process. This shift will be evident in a delay in eye fixations on the target word. Recall, in the visual world paradigm the listener is given time to review the four alternatives before the word is presented, and then asked to focus at the center of the monitor (where no picture is presented). Thus, these alternatives (top-down) can now compete for activation as the bottom-up auditory signal unfolds in time. When onset phonological competitors are presented, one can hypothesize that explicit processing will be activated. In these trials, two pictograms depicting words that share initial sounds (e.g., candle and candy) are presented on the monitor. As the spoken word unfolds in time, at least two alternatives are activated in response to input matching the pictograms. With more of the word heard, more information is accumulated and a mismatch can ensue between the bottom-up input and potential phonological alternatives, leading to explicit processing. Conversely, offset overlap competitors present less competition to the processing of the target word than onset overlap competitors (Allopenna et al., 1998). Thus, these trials should mostly lead to some degree of implicit processing. Increasing the working memory load from one to four digits might increase the competition generated by the shared final phonemes. We suggest that this increase in competition might shift speech processing from implicit to more explicit, delaying the onset of fixations on the target word. Note, explicit processing represents a slower processing of the spoken word, whereas implicit processing represents a faster one. When working memory load is low, the fast (implicit) processing of the initial sounds will minimize the impact of the shared offset sounds, as recognition might be reached earlier. However, when working memory load is high, the slower explicit processing will increase the competition presented by the offset sound sharing alternatives, as recognition is delayed. That is, one could hypothesize that increasing the load will have a larger impact on trials presenting offset overlap competition than on trials presenting onset overlap competition.

## The main experiment

We tested the role of working memory in the process of single spoken word recognition in ideal listening conditions. Young adults were tested both in high- and low-load conditions. We hypothesized that manipulating the load will have an impact on eye-fixations, especially in offset trials, that generally show only a small target-competitor competition for young good hearing individuals, when no load is utilized.

## Methods

### Participants

Twenty-four undergraduate students recruited from the Interdisciplinary Center (IDC) Herzliya, participated in the study in return for course credits. Their hearing thresholds were tested via a MAICO MA-51 audiometer. Four participants were excluded from analysis due to hearing impairments (PTA > 20 dB HL). Thus, 20 participants (*M* age = 24.2, *SD* = 2.0) were included in the analyses. All participants had pure-tone air conduction thresholds within clinically normal limits to their age range from 0.25 to 6 kHz in both ears (≤ 20 dB HL). Participants completed the Wechsler digit recall sub-task (WAIS IV, Wechsler, 2008), and their auditory working memory capacity was within expected values for their age range (*M* = 6.26, *SD* = 0.93). All participants were native Hebrew speakers, based on a self-report, and they achieved an average score on Wechsler subtest for vocabulary (*M* = 39.7, *SD* = 8.3) corresponding to above-average vocabulary levels for native Hebrew speakers (WAIS IV, Wechsler, 2008). All participants reported normal or corrected to normal vision, and when necessary, wore their own corrective eyewear.

### Paradigm construction

The current study adapted the “standard” visual world paradigm to Hebrew. Therefore, several preliminary steps were carried out to ensure that the basic paradigm yields comparable results in Hebrew.

### Visual stimuli

**Figure 1:**
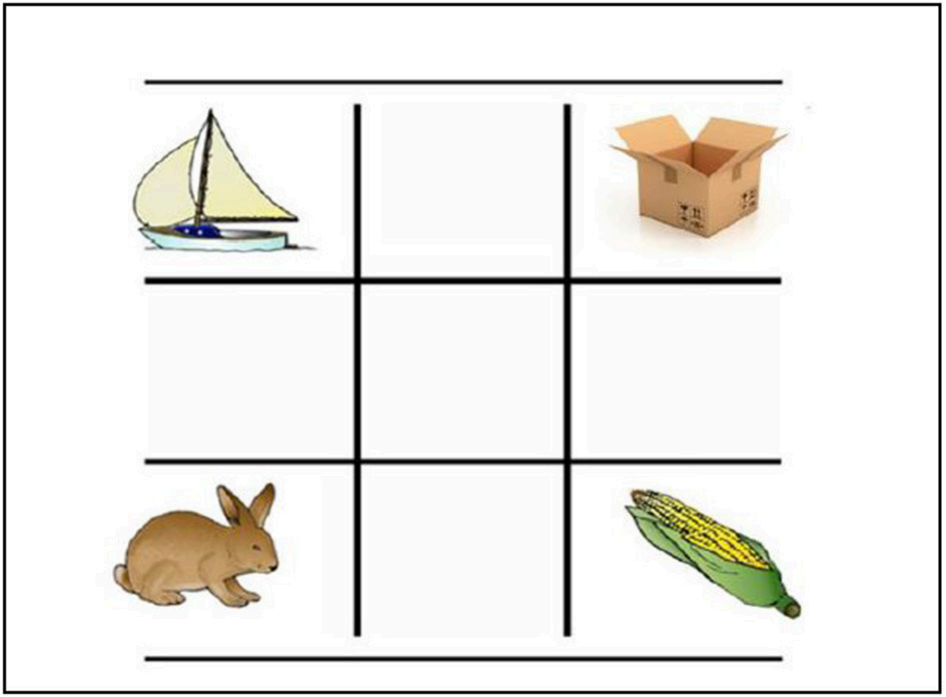
**Example of the experimental display in Hebrew**. The target word, “/aʁ. nav/” (bunny), is represented in the bottom left corner. The phonological competitor /”aʁ.gaz/” (box), is represented in the upper right corner “siʁa” and “tiʁas” (boat and corn, respectively) are unrelated fillers.

The experiment consisted of 32 critical trials (that include phonological competitors), 32 filler trials (that did not include phonological competitors), and eight practice trials. On all displays, four pictograms corresponding to object names in Hebrew were presented in the four corners of a 3 × 3 grid on a computer monitor (9 × 9 cm, subtending ~8.5° visual angle at a distance of 60 cm). We used a touch screen panel (T 23” ATCO infrared 4096 × 4096), to allow more natural response. We included only disyllabic words since in past research (Ben-David et al., 2011) disyllabic words yielded more accurate responses in a visual world paradigm. Images were not recycled in the critical nor in the filler displays, therefore 288 different images were used. The majority of images were drawn from the normed color image set of Rossion and Pourtois (2004). The remaining images were taken from commercial clip art databases and were selected to match the Rossion and Pourtois images in terms of visual style. In each critical trial, one pair of the depicted words either overlapped in the initial syllable (onset overlap) or in the final syllable (offset overlap). The critical trials summed to a total of 16 onset trials (e.g., /aʁ.gaz/ and /aʁ.nav/, box and bunny, respectively) and 16 offset trials (e.g., /xa.lon/ and /ba.lon/, window and balloon, respectively). In each critical trial, the target and its phonological competitor were presented alongside two unrelated stimuli that did not share onset- or offset-sounds with any of the words depicted in that trial. The relative position of pictograms within the grid (target, competitor, and two unrelated) was counterbalanced across the set of displays. An example of a critical trial is presented in Figure 1. Filler trials consisted of four pictograms that did not share onset- or offset-sound relations. The filler trials were included in order to diminish participants' expectations about the task and the phonetic semblance between the target and the competitor.

### Lexical items selection

In order to control for word frequency effects (Magnuson et al., 2007), we counterbalanced the target words in several ways. First, frequency of appearance in the language was measured by the Hebrew blog corpus (Linzen, 2009), based on a large corpus of blogs written in colloquial Hebrew. These frequencies were compared with the word frequency database for printed Hebrew in national newspapers (Frost and Plaut, 2005). Both databases used the orthographic form of the letter clusters, and were measured as the mean occurrence per million words. According to their frequencies, target words were equally distributed across the two experimental blocks, so that each block contained an equal number of the more frequent target words (which were counterbalanced across participants). Moreover, target–competitor allocation was counterbalanced as well, such that each word served for half of the participants as a target and for the other half as a competitor and vice versa.

### Image selection

To control for potential recognizability of display objects, 18 university students, native Hebrew speakers from the same population as our main experiment, were asked to name the critical images on an online questionnaire. Each image was presented for unlimited time. Fifty-nine out of the 64 experimental pictograms were highly recognizable (at least 75% name agreement). For the remaining five images, a different procedure was used, where participants were asked to rate: “to what extent (1–10) does the pictogram represent the word _____ [the object it is depicting].” This procedure was repeated with different images, until we found five pictograms that received scores higher than eight and these were included in the final set.

### Auditory stimuli

The stimuli consisted of the Hebrew equivalent of the sentence “point at the ____ [target word]” using the plural non-gender specific form (i.e., “/hats.bee.uh/ /al/ /ha/ [target word]”). These were prerecorded by a female native Hebrew speaking radio-actress in a professional radio studio (IDC radio), using a sampling rate of 48 kHz. The root-mean-square intensity was equated across all digitally recorded sentences, and the signal was played at 79 dB SPL. The average time interval between the onset of the recorded sentence and the onset of the target word was 1114 ms (*SD* = 97 ms), and the average noun duration was 1078 ms (*SD* = 91 ms) as measured from the recordings by three native Hebrew speakers using Praat software for analysis of speech (Version 5.4, Boersma and Weenink, 2004).

### Pre-test

The paradigm was validated in a pre-test with a group of participants taken from the same population as our main experiment. In the pre-test, we wished to validate the translation and other variations in the paradigm. For example, in the original paradigm, participants were instructed to move pictured objects (e.g., “put the apple that is on the towel in the box;” Tanenhaus et al., 1995). However, more recent research has used the instructions of looking at the target (e.g., “look at the candle,” Ben-David et al., 2011) or clicking on it with a computer mouse (e.g., Allopenna et al., 1998) for selection of the objects. As the former instructions might provoke more conservative eye movements and the latter might be less direct, we used a set-up that allowed us to collect responses by a touch screen. Thus, participants were simply asked to point with their finger at one of the objects on the monitor (e.g., “point at the candle”). The results of the pre-test confirmed that the baseline paradigm in Hebrew generates similar eye fixations patterns as previous findings (e.g., Ben-David et al., 2011).

### Procedure

Participants were tested individually in a single walled sound attenuated booth (IAC). They were seated at a distance of 60 cm from the computer monitor, resting their chin on a chin rest. Eye movements were recorded via a table-mounted eye tracking system (SR Research Eyelink 1000, using the “tower mount” configuration), which sampled eye gaze position every 2 ms. Each block of trials began with a calibration procedure followed by four practice trials. Within each block, 16 critical trials (eight with onset overlap and eight with offset overlap) were pseudo randomly interleaved with 16 filler trials, with the exception that the first four trials were always fillers. Participants completed two blocks, high- and low-load (counterbalanced). In the high-load block, four random digits were played prior to the speech perception task, at a pace of one digit per second. The digits were prerecorded by the same female actress that read the instructions. Participants were asked to retain these digits for later recall. In the low-load block, participants were presented with only one random digit for later recall. Each trial began with a visual cue (black triangle on a white background) immediately followed by the auditory presentation of the digits. Then, a 3 × 3 grid appeared on the monitor, containing the four pictograms at each corner of the grid. After 2000 ms, a short 1-kHz tone was played, directing participants to focus on the fixation cross which simultaneously appeared in the center of the grid.

After the system registered cumulative fixations on the central square for at least 200 ms, the fixation cross disappeared, and the recorded instruction sentence was played. Participants were instructed to point at one of the four objects on the monitor. A choice was indicated by touching the pictogram on the monitor. A feedback signal followed the participant's choice; either a green square (denoting “correct”) or red (“incorrect”) masked the cell. The feedback was administered in order to attain the highest degree of accuracy and attention for the whole duration of the task.

The objects then disappeared from the grid to signal the end of the trial, and a visual cue (black circle on a white background) was presented, indicating recall. Participants were instructed to report the digits verbally in the order in which they had been presented. Instructions emphasized that performance on both tasks were equally important. At the end of the procedure, participants were probed for whether they suspected a connection between the pictograms and were debriefed.

### Interest areas

Interest areas were defined in rectangular regions around each image, following the grid. Interest areas were also defined for each of the remaining five regions of the grid as well as off-screen, but these were not included in the subsequent analysis. The samples were then grouped and binned into 20 ms time-bins, with 10 samples summed per bin. Data retained for each time-bin included the target fixation count (i.e., the number of samples per bin that contained a fixation on the target).

## Statistical analysis

### Eye-movements analysis: fixations on the phonological competitor

We tested whether aggregated fixations on the phonological competitor (total time fixating on the competitor, see Helfer and Staub, 2014) were significantly higher than average aggregated fixations on the unrelated nouns (from 200 to 1500 ms after the onset of the word). We used a repeated-measures ANOVA, with the type of noun (phonological competitor vs. unrelated noun), type of overlap (onset vs. offset), and load (high vs. low) as within participant factors. We found a main effect for the type of noun [*F*_(1, 19)_ = 9.89, *p* = 0.005, η^2^ = 0.34], indicating that, overall, phonological competitors generated more fixations than the unrelated nouns (averages of 3.5 and 2.5% of possible fixations, respectively)—showcasing the competition on processing. No significant main effects were found for the type of competitor, for load, and none of the two or three-way interactions were statistically significant (*p* ≥ 0.09, for all). This indicated that neither of these factors nor the interactions between them had an impact on fixations on items other than the target. As a consequence, fixations on the phonological competitors will not be further discussed.

### Modeling eye-movements analysis: fixations on the target word

Analyses were made on trials in which the digits were correctly retained. Once a selection was made (by pressing on the correct pictogram), we considered all the following time bins as fixations on the target (applying the same procedure as in Ben-David et al., 2011). This facilitated the comparison of different trials, independent of the amount of time taken by the participant to select the target. Note, at the time they make a selection, participants have already reached a decision about the spoken word. Thus, we opted to use a cumulative approach—where we report, at each time bin, the percent of trials where the participant had reached recognition of the target word.

We used Mirman's Growth Curve Analysis (Mirman, 2014), which is a multilevel regression technique designed for time course analysis, and specifically to the visual world paradigm. This method was chosen as it utilizes the fine-grained data eye-tracking provides, while avoiding the power-time-resolution tradeoff^1^. Three orthogonal time-vectors were computed from the time data. These vectors corresponded to first, second, and third-degree time terms, to help isolate the different polynomial time effects of the model parameters. We applied a mixed-effects model containing fixed effects of the competitor overlap (onset vs. offset), the working memory load (low vs. high), and the combined effect of the two on the intercept and all three time-terms. Random effects of the participants on the intercept and each time-term were also included. The mean of the model's predicted response was then plotted for each combined level of the factors. The overall time course of target fixations (from word onset to 2980 ms after word onset) was captured with a third-order (cubic) orthogonal polynomial with fixed effects of condition (low vs. high load) on all time terms, and participant and participant-by-condition random effects on all time terms. The low-load onset competition model was treated as the reference (baseline) and relative parameters estimated for the remaining three models (onset-high load, offset-low load, and offset-high load). For the models, time bins of 20 ms were used (10 samples per time bin, and 50 time bins per second), providing 125 measurements per trial in the period of interest, (for details, see Mirman, 2014). Statistical significance for individual parameter estimates was assessed using the normal approximation. Specifically, because the high-resolution time-course data provided us with relatively many measurements, we assumed the *t*-scores calculated for the coefficient estimators were normally distributed and approximate *z*-values. All analyses were carried out in R statistical software (version 3.1.3). The lme4 package (version 1.1–10) was used to fit the linear mixed-effects models. All R packages were downloaded from the CRAN package repository (R Core Team, 2016).

## Results and discussion

### Accuracy analysis

(a) *Target selection*. The target spoken word was correctly selected (100% accuracy) in all trials in both high and low load conditions. (b) *Digit recall task*. The mean accuracy across all conditions for the digit recall task was very high (*M* = 98.3%, *SD* = 4.1). However, it was slightly better for the low-load (one digit) relative to the high-load (four digits) condition (99.7 vs. 96.9%). Yet this difference was not found to be significant in a repeated measures ANOVA of digit-span accuracy with type of competitor (onset or offset) and working memory load (high or low) as within-participant factors.

### Eye-movements analysis—fixation on the target word

**Figure 2:**
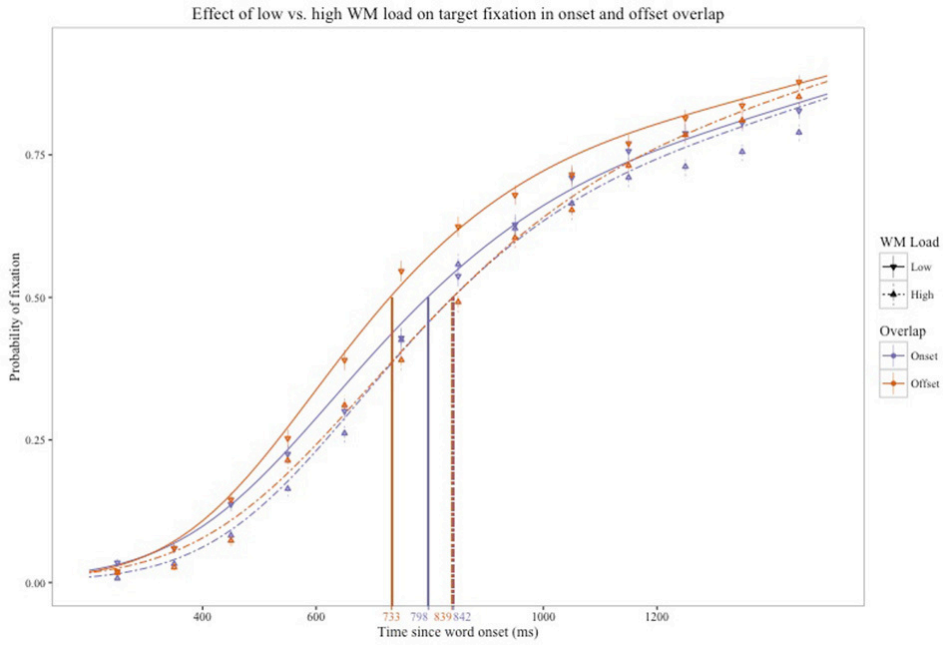
**Fixation proportions for the target words in onset and offset competition trials, from 200 to 1500 ms since word onset**. Continuous lines represent a growth curve model for low working memory load and the dashed lines for high working memory load. Orange and purple lines represent the offset- and onset-competition trials, respectively. Vertical lines represent the 50% threshold for the four models.

Figure 2 presents the data and the model for the offset (orange line) and onset (purple line) competitor trials, in the low load (continuous line) and the high load (dashed line) conditions.

First, all coefficients of the base model, onset low load, were found to be significant (see Appendix 1 in Supplementary Material), indicating that the model presents a good fit to the data. Second, this base model was compared to the other three models (onset-high, offset-low, offset-high). All parameters of the other three models were significant (linear, quadratic, and cubic)^2^.

To estimate the main effects of load (high vs. low), type of competition (onset vs. offset) and the interaction of the two on the model, Chi-square tests were conducted (see, Appendix 2 in Supplementary Material). There was a significant effect of working memory load, indicating that the models for low load were different from the models representing high load conditions across onset and offset phonological competition. Specifically, as indicated by observing Figure 2, the models for the high load conditions show slower accumulation of information. To exhibit this effect of load, Table 1 presents the thresholds for 25, 50, and 75% recognition in ms (the points in time after which the chance of fixating on the target was above 25, 50, or 75%) based on the model estimation. Note that across the three thresholds, the recognition in the high load conditions occurs later than in the low load conditions.

**Table 1:** **Thresholds in ms for 25, 50, and 75% recognition, based on the model, as a function of the type of phonological (onset vs. offset) overlap and load (high vs. low)**.

	**Onset**		**Offset**	
	**High load**	**Low load**	**High load**	**Low load**
25%	616	567	606	534
50%	842	798	839	733
75%	1226	1180	1176	1060

The data also show an effect of phonological overlap, where the models for onset were different from the models for offset competition (see Appendix 1 in Supplementary Material). Finally, a significant interaction of the two main effects was found. Examining Figure 2, it appears that the interaction reflects a larger effect of load on offset compared to onset competition. This differential effect of load is also evident by examining the model based thresholds in Table 1. Consider the 50% threshold for target recognition. Load delayed the threshold by 44 ms in onset competition trials and by 106 ms in offset competition trials. In sum, a four-digit preload delayed fixations on the target, but to a larger degree when the display presented offset-overlap competition.

## General discussion

The goal of the current study was to examine the influence of working memory load on spoken word recognition. Load was manipulated by retaining either four spoken digits (high load) or one digit (low load). By monitoring eye-movements, we were able to reveal a delay of more than a 100 ms in the activation of the spoken target word (50% threshold in offset competition). Notably, listening conditions were ideal, and accuracy rates were at ceiling, indicating participants' adherence to the instructions and the ease of the task. Not only was the speech recognition task easy, but also the digit recall task, as participants' average working memory capacity (as tested before the study) was substantially larger than four digits. Nevertheless, even though no extreme boundaries were reached, and the additional load had no effect on accuracy, we were still able to observe a slowdown in the recognition process due to working memory load.

### Offset vs. onset competitor

Examining fixations on competitors, our data are consistent with evidence from continuous mapping models (e.g., TRACE; McClelland and Elman, 1986), where both onset and offset competition play a role in spoken word recognition. Across conditions, we found that the time spent fixating on the phonological competitors was, on average, higher than the time spent fixating on the unrelated items.

Turning to target fixations, we note a main effect for load, with delayed fixations in the high load condition, and a main effect for the type of phonological overlap with delayed fixation for onset competition. The latter result supports previous works demonstrating weaker activation for offset relative to onset competition in young good hearing adults (Allopenna et al., 1998; Tanenhaus et al., 2000; for supportive data from gating studies on onset vs. offset competition see Wingfield et al., 1997). Moreover, the size of the digit pre-load was found to have a larger impact on target recognition with offset competition compared to onset competition. In other words, increasing the pre-load from one to four spoken digits was sufficient to produce a prominent competition from offset-sound sharing alternatives, as reflected by a slowdown in target fixations function. This can relate to a reduced ability in the high-load condition to efficiently inhibit the processing of offset alternatives, which might be easily discarded in the low load condition (Lavie et al., 2004).

Our results may also suggest that in the high-load condition, listeners were slow on the uptake of the spoken word (sluggish onset). For example, when the offset sharing pair /xa.lon/—/ba.lon/ (window-balloon) is presented, slower processing of the initial sounds (that distinguish between the two alternatives) would increase the competition generated as the shared /lon/ sound unfolds. However, theoretically, this slowed processing of initial sounds should not increase competition at the onset of the word (e.g., /ar.nav/—/ar.gaz/; for a discussion on applying information theory to the analysis of signal processing, see Ben-David and Algom, 2009).

This slowdown in the processing of the initial sounds of the word is in line with the hypothesis that when working memory demands are higher (fewer resources are available), it takes longer for the speech sound stream to form into an auditory object (Kubovy and Van Valkenburg, 2001; for a review see, Griffiths and Warren, 2004). In such a case, integrating the phonemes into a coherent object (word) might have been delayed due to the working memory load. As a result, listeners were slower to process the initial sounds of the word. Moreover, Sörqvist et al. (2012) noted that an increase in working memory load was related to a decrease in a very early auditory sensory processing stage (measured by auditory evoked brain stem responses, ABR). However, the auditory stimuli were not at the center of listeners' attention nor were they speech-like. Clearly, more research is needed to examine whether the formation of auditory objects is impacted by load when speech is presented in quiet and there is no need to segregate streams.

The sluggish onset of word processing may also relate to the working memory load task itself. The phonological loop in the Baddeley model (Baddeley and Hitch, 1974; Baddeley, 1986) is the mechanism for temporary storage for phonemic information, and when it is occupied, the processing of other auditory information is impaired (e.g., Burgess and Hitch, 1999). This might suggest that the phonological loop, being preoccupied with rehearsing the preloaded digits, is responsible for the delay in word processing. It is possible that processing of the initial sounds of the word was hampered until the digits were encoded into long-term memory (LTM). Transferring the digits to LTM “freed” the phonological loop, enabling it to process effectively what is retained of the word (for a similar notion, see Rönnberg et al., 2013).

### Relating our data to aging research

It is possible to consider the links between our results in the high working memory load condition and the data obtained in similar studies with older adults. As older adults have reduced working memory capacity (Zacks, 1989; Salthouse et al., 2003; Gazzaley et al., 2005; Small et al., 2011), one may claim that performance in the high-load task can somewhat simulate the reduced working memory capacity indicated in older adults. Comparing our data to Ben-David et al. (2011) data shows interesting similarities between the processing of older adults, and the processing of younger adults in the high load conditions. The authors found substantially larger age-related effects on processing in the offset overlap condition than the onset (see Figures 6A,B, p. 253, Ben-David et al., 2011). The authors explained this difficulty in offset as the consequence of older adults' less synchronized matching of auditory input to the mental lexicon, potentially due to reduced working memory capacity. It is possible that the working memory load manipulation might have a similar impact on our participants' speech processing, by decreasing available resources for recognition. Further research can use the same working memory manipulation on older adults and observe whether offset competitor processing deteriorates more than onset.

### Relating our data to the ELU model

The ELU model (Rönnberg et al., 2013) posits that when there is a good match between the bottom-up acoustic input and the corresponding phonological representation in LTM, speech is processed implicitly with little or no demands on working memory resources. Further, task difficulty determines the allocation of resources to explicit speech processing that may include cognitive functions such as inhibition, executive functions, and working memory (McCabe et al., 2010). When the competition between bottom-up and top-down information increases, a shift is expected from implicit to more explicit processing. In our data, this shift might be reflected by a delay in gaze fixations on the target. We suggest that explicit processing for onset overlap (where competition is greater) was already employed in the low load condition. Thus, the increase in working memory load affected to a lesser degree the processing or gaze fixations for onset overlap in high load. Offset overlap trials, on the other hand, generated relatively little competition in the low load condition, and thus mostly relied on implicit processing. In the high-load condition, the additional demands on working memory amplified the competition, triggering the engagement of explicit processing. This was reflected in a delayed 50% threshold for gaze fixations on the target word.

## Future studies

Future studies should further investigate how aging and background noise can impact the role of working memory in speech processing. One of the biggest difficulties older adults have to cope with is deteriorated speech comprehension, especially in noisy environments (Schneider et al., 2010) and with increased demands (see Wingfield et al., 2015). This difficulty can interfere with maintenance of health and quality of life (Ishine et al., 2007; Gopinath et al., 2012) and can potentially affect the rate of cognitive decline (Lin, 2011). A central research question in speech recognition in older adults is the extent to which difficulties stem from bottom-up, sensory declines that degrade the speech input (Schneider and Pichora-Fuller, 2000), and to what extent they stem from an age-related decline in working memory (e.g., Bopp and Verhaeghen, 2007) and related cognitive abilities (e.g., inhibition of irrelevant distractors, see Ben-David et al., 2014; Lash and Wingfield, 2014). Specifically, a recent study may suggest that an increase in task demands (shifting from noise to babble background) hampered the ability of older adults to quickly generate independent target-word and background auditory streams (Ben-David et al., 2012). We hope that by adapting the paradigm used in the current study to test an older adult population, one can learn more about the role of working memory in speech processing in older age. Finally, more work is called for in Hebrew to see whether the language and the associated culture may contribute to the discussed effects. One such factor may be changes in the rate of speech across cultures and languages (see Ben-David and Icht, 2015; Icht and Ben-David, 2015), or unique attributes of Hebrew itself (e.g., the role of consonantal roots, see Frost et al., 1997).

## Author contributions

BH is responsible of the design of the paradigm, had prominent intellectual contribution, approval of the draft, and accountable for the data. JS is responsible for the analysis and interpretation of the data, revising it, had intellectual contribution, approval of the draft, and accountable for the data. AW had intellectual contribution of interpreting the results, approval of the draft, and accountable for the data. BB is responsible of the design of the paradigm, the analysis and the interpretation of the results. Had prominent intellectual contribution, approval of the draft, and accountable for the data.

### Conflict of interest statement

The authors declare that the research was conducted in the absence of any commercial or financial relationships that could be construed as a potential conflict of interest.

## Footnotes

^1^Mirman (2014; Mirman et al., 2008; Britt et al., 2014) discussed three main challenges of analyzing visual world time course data using *t*-tests or ANOVA. (1) Trade-offs between power and resolution. Namely, as each time-bin has limited data, we need larger time-bins to increase statistical power. Yet, this will reduce temporal resolution and thus valuable information on the gradual change over time might be lost. (2) Possibility of experimenter bias. In the traditional analyses, the experimenter must choose the time bin size, and the time boundaries for the ANOVA. These choices might introduce a bias. (3) Statistical thresholding. The time-bin by time-bin tests, must treat p values that are < 0.05 as fundamentally different as those above 0.05. Thus, small noisy changes in the data may lead to over- or under-estimation of discreet differences.

^2^Except of the intercept of onset high load, which was not significantly different than the intercept of the base model (see Appendix 1 in Supplementary Material).
