# Supplementary material for: Working Memory Load Affects Processing Time in Spoken Word Recognition: Evidence from Eye-Movements
Source: Front Neurosci. 2016 May 19;10:221. doi: 10.3389/fnins.2016.00221 (PMC4871876; doi:10.3389/fnins.2016.00221)
Supplement: Supplementary file 1 [file DataSheet1.docx]

**Appendices**

|  | Time term | Estimate | Std. Error | Z-value | p |
| --- | --- | --- | --- | --- | --- |
| Base model - Onset low-load condition) | Intercept | 3.71 | 0.74 | 5 | < .001 |
|  | Linear | 74.68 | 11.99 | 6.23 | < .001 |
|  | Quadratic | 18.19 | 8.05 | 2.26 | 0.02 |
|  | Cubic | 18.29 | 4.2 | 4.35 | < .001 |
|  |  |  |  |  |  |
| Deviations for the Onset- high load condition | Intercept | -0.01 | 0.02 | -0.34 | 0.73 |
|  | Linear | 5.86 | 0.35 | 16.94 | < .001 |
|  | Quadratic | 0.76 | 0.26 | 2.96 | < .001 |
|  | Cubic | 2.15 | 0.26 | 8.4 | < .001 |
|  |  |  |  |  |  |
| Deviations for the Offset- low load condition | Intercept | 0.93 | 0.03 | 28.66 | < .001 |
|  | Linear | 12.86 | 0.6 | 21.52 | < .001 |
|  | Quadratic | 6.89 | 0.42 | 16.32 | < .001 |
|  | Cubic | 4.54 | 0.33 | 13.93 | < .001 |
|  |  |  |  |  |  |
| Deviations for the Offset- high load condition | Intercept | -0.84 | 0.04 | -21.77 | < .001 |
|  | Linear | -16.3 | 0.7 | -23.35 | < .001 |
|  | Quadratic | -8.83 | 0.5 | -17.6 | < .001 |
|  | Cubic | -8.42 | 0.42 | -20.03 | < .001 |

**Appendix 1**. Coefficients of the model of target fixation proportions in overlap trials.

|  | Time term | Chi-square | p |
| --- | --- | --- | --- |
|  | Intercept | 839.56 | < .001 |
|  | Linear | 7.69 | < .001 |
|  | Quadratic | 6.68 | < .001 |
| Overlap | Cubic | 7.13 | < .001 |
|  |  |  |  |
| Load | Intercept | 1098.75 | < .001 |
|  | Linear | 36.33 | < .001 |
|  | Quadratic | 54.5 | < .001 |
|  | Cubic | 24.31 | < .001 |
|  |  |  |  |
| Overlap X load | Intercept | 170.83 | < .001 |
|  | Linear | 545.37 | < .001 |
|  | Quadratic | 309.69 | < .001 |
|  | Cubic | 401.32 | < .001 |

**Appendix 2**. Chi-square tests for the effects of overlap (onset vs. offset), load (high vs. low) and their interaction.
